# Cueing Paradigms to Improve Gait and Posture in Parkinson’s Disease: A Narrative Review

**DOI:** 10.3390/s19245468

**Published:** 2019-12-11

**Authors:** Niveditha Muthukrishnan, James J. Abbas, Holly A. Shill, Narayanan Krishnamurthi

**Affiliations:** 1 Center for Adaptive Neural Systems, School of Biological and Health Systems Engineering, Arizona State University, Tempe, AZ 85287, USA; Niveditha.Muthukrishnan@asu.edu (N.M.); James.Abbas@asu.edu (J.J.A.); 2 Muhammad Ali Parkinson Center, Barrow Neurological Institute, St. Joseph’s Hospital and Medical Center, Phoenix, AZ 85013, USA; Holly.Shill@DignityHealth.org; 3 Edson College of Nursing and Health Innovation, Arizona State University, Phoenix, AZ 85004, USA

**Keywords:** Parkinson’s disease, cueing, gait, posture, rehabilitation, wearable sensors

## Abstract

Progressive gait dysfunction is one of the primary motor symptoms in people with Parkinson’s disease (PD). It is generally expressed as reduced step length and gait speed and as increased variability in step time and step length. People with PD also exhibit stooped posture which disrupts gait and impedes social interaction. The gait and posture impairments are usually resistant to the pharmacological treatment, worsen as the disease progresses, increase the likelihood of falls, and result in higher rates of hospitalization and mortality. These impairments may be caused by perceptual deficiencies (poor spatial awareness and loss of temporal rhythmicity) due to the disruptions in processing intrinsic information related to movement initiation and execution which can result in misperceptions of the actual effort required to perform a desired movement and maintain a stable posture. Consequently, people with PD often depend on external cues during execution of motor tasks. Numerous studies involving open-loop cues have shown improvements in gait and freezing of gait (FoG) in people with PD. However, the benefits of cueing may be limited, since cues are provided in a consistent/rhythmic manner irrespective of how well a person follows them. This limitation can be addressed by providing feedback in real-time to the user about performance (closed-loop cueing) which may help to improve movement patterns. Some studies that used closed-loop cueing observed improvements in gait and posture in PD, but the treadmill-based setup in a laboratory would not be accessible outside of a research setting, and the skills learned may not readily and completely transfer to overground locomotion in the community. Technologies suitable for cueing outside of laboratory environments could facilitate movement practice during daily activities at home or in the community and could strongly reinforce movement patterns and improve clinical outcomes. This narrative review presents an overview of cueing paradigms that have been utilized to improve gait and posture in people with PD and recommends development of closed-loop wearable systems that can be used at home or in the community to improve gait and posture in PD.

## 1. Introduction

Parkinson’s disease (PD), which is the second most common progressive neurodegenerative disease, results in motor and non-motor dysfunctions caused by the degeneration of dopamine-producing cells of the substantia nigra and other brain regions [1,2]. Clinical motor symptoms include bradykinesia, tremor, rigidity, freezing of gait, and instability of posture and gait [3,4,5,6]. Some of the common manifestations of PD that affect gait and posture are stooped posture and shuffling of gait, increases in gait asymmetry and double support time, reductions in step length and walking speed, impairments in postural responses to perturbations, and increases in variability of step/stride time as well as step/stride length [7]. Considerable efforts are being taken to improve options for treating mobility deficits in persons with PD because of the associated risk of falls and loss of independence.

Pharmacological and deep brain stimulation (DBS) surgical treatments have been demonstrated to be partially effective in managing some of the manifestations of gait impairments and postural instability. As the primary pharmacological treatment in PD, the dopamine replacement therapy (i.e., levodopa) improves stride length, gait speed, and double support time variability, whereas it does not have any significant benefits on cadence and other temporal characteristics of gait [8]. The effects of levodopa on postural sway is controversial [9,10]. Regarding inadequate postural responses (compensatory stepping) leading to falls in PD, levodopa seems to offer no benefit [11,12]. Thus, the effects of levodopa on gait and posture in PD is inconsistent.

Concerning the effects of the DBS, stimulation of subthalamic nucleus (STN-DBS) consistently improved stride length but no effects on stride time and its variability were found. Stimulation of globus pallidum internus (GPi-DBS) significantly improved gait velocity but without any significant improvements in stride length. Also, many people with PD reported postoperative worsening of gait and increased risk of falls [13]. In the case of the stimulation of pedunculopontine nucleus (PPN-DBS) at 15–70 Hz, improvements in postural instability and gait disorder, including freezing of gait and falls, have been noticed. However, the improvements varied depending on the duration of follow-up and types of outcome measures obtained [14]. Low-frequency STN-DBS and GPi-DBS (below 100 Hz) have shown encouraging beneficial effects on axial symptoms in PD; however, higher levels of evidence with randomized and blinded studies are needed to confirm the benefits [15]. Also, the overall benefits of low-frequency STN-DBS decrease with long-term use [16].

## 2. Pathophysiology of Motor Dysfunction in PD

**Figure 1:**
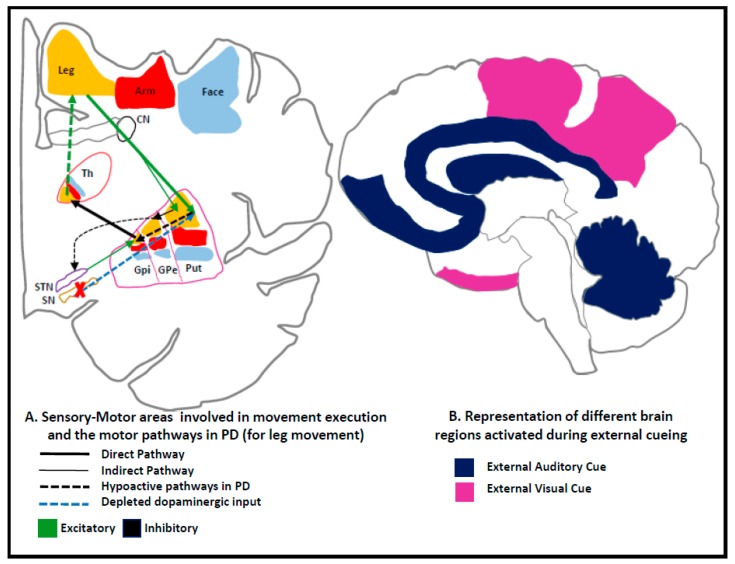
(**A**) Sensory-motor areas for movement execution in the basal ganglia and the impaired motor pathways in Parkinson’s disease (PD) with the prevalence of the indirect pathway over the direct pathway and the affected SN’s input to the circuit. SN—Substantia nigra, GPi—globus pallidus internus, GPe—globus pallidus externus, Put—putamen, Th—thalamus, CN—caudate nucleus, STN—sub-thalamic nucleus. This results in increased neuronal firing activity in the output nuclei of the basal ganglia that leads to excessive inhibition of thalamo-cortical and brainstem motor systems which, in turn, interferes with movement onset and execution [28,29]. (**B**) Representation of brain areas activated during external cueing reported from findings of image analysis studies conducted on people with PD during cueing experiments [17,30,31,32].

The loss of dopaminergic neurons in the substantia nigra pars compacta within the basal ganglia leads to classical parkinsonian motor symptoms. The basal ganglia play significant roles in the production and control of automatic and well-learned motor movements. First, the basal ganglia generate internal cues or trigger to facilitate the initiation of movement sequences without attention. Second, they contribute to the cortical “motor set”, i.e., they aid in the preparation and maintenance of motor schemes in a state of action readiness thereby enabling appropriate motor function execution. The widely accepted model of basal ganglia consists of two circuits, the direct and indirect pathways, which originate from striatal neurons and project to various output structures. The direct pathway is postulated to promote movement by direct inhibitory projections to the globus pallidus internus/substantia nigra reticulata (GPi/SNr), whereas the indirect pathway is hypothesized to inhibit movement projecting to the GPi/SNr through globus pallidus externus (GPe) and subthalamic nucleus (STN). In PD, striatal dopaminergic depletion results in the reduced inhibitory direct pathway and increased indirect pathway output onto the GPi/SNr and, subsequently, increased GPi/SNr inhibition to the output structures. This consequently leads to deficiencies in the execution of a movement [6,17,18,19] (Figure 1). This deficiency in execution results in hypokinesia, a central feature in PD, or lack of movement together with muscular rigidity.

Evidence indicates that the basal ganglia are also important for sensorimotor integration. Striatal cells are robustly activated when a sensory event functions as a cue for a movement. In addition, the caudate nucleus and substantia nigra contain a large proportion of cells that are multisensory; such cells could be used to integrate sensory inputs and form a multimodal representation of the environment in the basal ganglia. Disruption of basal ganglia processes enhances the response of pallidal neurons to passive limb movement, suggesting an impaired gain mechanism because of dopamine depletion [2,4,20]. A common consequence of striatal dopamine loss is attenuation of the transfer of critical information to the basal ganglia which leads to a decrease in the ability to detect relevant internal sensory and or movement cues [1,21,22]. Such a disruption of information flow to the basal ganglia may worsen impaired movement selection and sequencing in striatum with dopamine loss thereby resulting in gait impairments [23]. The pattern of deficits in people with PD is consistent with a disruption of this integration mechanism. Persons with PD may become increasingly dependent on external stimuli to initiate and shape motor output and may be unable to effectively execute movements because of the lack of critical proprioceptive information [24,25,26,27].

The presentation of cues in PD is hypothesized to compensate for the pathology by increasing cortical activation which diminishes pathological activity (10–30 Hz) in the basal ganglia [33], mainly by suppressing the subthalamic nucleus through direct pathways [34]. In the case of visual cues, the unaffected visual-motor pathways are believed to play a major role in facilitating movements bypassing the basal ganglia [35].

## 3. Methodology

In this review, the current state of scientific knowledge associated with cueing to improve gait and posture in PD is presented. The search for research articles involving cueing/feedback to improve gait/posture in PD used combinations of the following keywords: Parkinson’s disease, cueing/cues/cue, real-time feedback, gait, and posture. From the set of 304 articles returned by the search, only studies that used quantitative gait and posture outcome measures (e.g., step length, stride length, walking speed, cadence, and posture) were included. The set of studies were then categorized by type of feedback implemented (visual, auditory, somatosensory), by wearability/non-wearability of the cueing device/mechanism, and by study duration (single-session or long-term training). References cited in the selected publications were also examined for other relevant studies to be considered. Studies were excluded if they were not directed for people with PD, did not measure spatiotemporal parameters of gait and/or posture, or used non-cue-based gait and posture rehabilitation strategies.

## 4. Cueing for Rehabilitation in PD

Given the limited ability of pharmacological and surgical treatments to address gait and postural impairments in PD, various forms of external cueing (visual, auditory, or somatosensory) are being investigated for inclusion in neuromotor rehabilitation programs. Cueing can be defined as a mechanism of applying a spatial or a temporal stimulus to facilitate initiating or maintaining motor activity [32]. Numerous studies have shown that external cueing can improve the amplitude and timing of the intended movement by increasing body position/movement awareness, making it a suitable modality for gait and posture rehabilitation [25,26,36,37,38,39,40]. In addition, cueing has also been increasingly used in helping with the initiation of a movement [41].

Cueing studies could be classified as open-loop cueing or closed-loop cueing based on how the cue is presented. In open-loop cueing, the user is presented a series of cues in a periodic or preset manner that is independent of the user’s performance. Metronome beats and a set of lines on the floor separated by a preset distance are examples of open-loop temporal and spatial cues, respectively. Open-loop studies have most widely utilized auditory or visual forms of cues to improve gait in people with PD. While auditory cues have most often been delivered as rhythmic auditory stimulation (RAS) or metronome beats in accordance to the user’s preferred gait speed or cadence [36,42,43,44,45,46], other types of cues such as highly rhythmic music or verbal instructions have also been investigated in some of the studies [43,44,47]. Most forms of visual cueing present lines or markers on the floor as targets for foot placement. Markers such as stripes/tapes on the floor, projections from laser pointers, and lights mounted on the user or embedded on a walking stick or walker [48,49,50,51,52] have been utilized. Visual cues were spaced at distances based on the subject’s average step/stride length measured at baseline trials. A few studies have investigated the use of somatosensory cues [53,54,55] using vibrating wrist-worn devices and a combination of audio/visual and or/somatosensory cues for rehabilitation [40,56,57].

Studies of open-loop cueing used as a therapeutic modality have demonstrated short-term and long-term gait improvements [41,42,43,48,58,59,60,61]. Short-term studies investigated immediate effects with and without different cue interventions [62,63,64]. Laboratory-based long-term training studies compared walking with cues to without cues [50]. One long-term auditory cueing study investigated differences between ecological-based footstep cues (sound recorded while walking on gravel) to artificially synthesized RAS [43] and compared walking with auditory cues to walking with visual cues [40]. In the studies that presented cues as training, cues were provided progressively [65] or in combination with physical therapy improved step time variability [59], posture and bradykinesia [66], stride length, gait speed, and cadence [42].

In contrast with open-loop cues, closed-loop cueing provides feedback on the user’s performance in real-time which can facilitate modifying one’s performance to achieve the desired movements. Real-time feedback of step length [67,68,69,70] and uprightness of posture [69] have been investigated for targeting PD-specific gait and posture deficits. However, these studies used treadmill-based cueing systems and, therefore, are not suitable for overground locomotion during free-living conditions.

Many studies have been performed using virtual reality (VR) which provides visual stimuli that can help in motor and cognitive training [60,62,71,72,73,74,75,76]. These studies have used augmented visual/auditory- or somatosensory-based feedback for training, but a meta-analysis [77] indicated that there is only limited evidence of improvements in gait and balance due to the use of VR compared to an active intervention without the VR component. Importantly, most of these VR systems require a very sophisticated and expensive setup and may not be suitable for use at home.

## 5. Benefits of Open-Loop Cueing on Gait in PD

Evaluation of the acute/immediate effects of cues demonstrated that gait variables, such as cadence [48,52,56,59,78,79], speed [48,52,57,59,80], and step length [42,44,45,48,63,80], increased during walking with rhythmic auditory stimulation (RAS) when compared to walking without cues. In some instances, the improvements in step length were reported to be a consequence of using a cadence that was higher than the baseline. In addition to improving stride length and temporal measures, RAS also reduced stride-time variability [81] and helped persons without freezing of gait (FoG) more than those with FoG [79]. It was suggested that RAS might provide an external rhythm that can compensate for the defective internal rhythm of the basal ganglia in PD [45,50,80].

Use of visual cues, on the other hand, consistently improved step/stride length [49,50,51,70,71,73] with or without increasing walking speed or cadence. Plausible explanations for these acute effects could be that visual cues may help fill in for the motor set deficiency by providing visual-spatial data [17,82] and help in focusing attention on gait [57,73]. However, in studies that involved visual cueing during treadmill walking, it is not clear whether the gait benefits were due to the visual cueing or to the external pacemaker effect of the treadmill. Also, treadmill walking at speeds greater than the comfortable speed may demand more attention to the task of walking itself, which may result in worsening gait automaticity (ability to perform upper and lower limbs movements automatically during gait with little attention) which is already reduced in PD compared to age-matched controls [52,83]. An investigation of a visual cueing strategy that used a subject-mounted light device to present step length cues at a preset distance in front of the user reported improvements in stride length and gait speed [49]. Cueing studies that combine auditory, visual, or somatosensory cues [40,56] also reported improvements in cadence, gait speed, and stride length. Moreover, studies that focused on attention strategy by asking people with PD to think about taking larger strides were found to be effective in normalizing gait deficits observed in PD [47,57].

Notably, studies that have investigated the impact of long-term training demonstrated that RAS was effective in improving both temporal and spatial gait measures, such as walking speed, cadence, and step/stride length, regardless of the type of sound stimulation (ecological, synthetic auditory cue) that was provided. A follow-up evaluation conducted after three months revealed that the effects of the training were still largely maintained. When RAS was used for one-week training in PD people with FoG [58], walking speed was increased, but no change in freezing episodes was noted, whereas, in another study that used RAS for a three-week training, stride length, walking speed, and cadence were significantly increased [42]. Effects of long-term gait training with and without visual cues showed increases in step length and gait speed [50]. An open-loop cueing study that demonstrated improvements in both temporal gait parameters and stride length attributed temporal improvements with the use of auditory cues and improved stride length to the visual cues [40]. Results from a similar study [66] showed improvements in postural stability and bradykinesia (as measured using Unified Parkinson’s Disease Rating Scale (UPDRS)-Part III items) that were retained six weeks after the training period was completed.

## 6. Benefits of Closed-Loop Cueing on Gait in PD

Closed-loop cueing provides feedback based on the user’s movements in real-time so that the user can be aware of their performance and modulate it to achieve the desired/target performance. Studies that investigated closed-loop cueing are fewer in number and are more recent as compared to open-loop strategies. Both single-session and long-term training studies using closed-loop cueing were conducted using auditory, visual, somatosensory, and combined cueing strategies to evaluate their effects on gait and posture. Studies that used closed-loop feedback systems have demonstrated a higher degree of gait and posture improvement as well as residual carry-over effects in comparison with open-loop, feed-forward systems [39,81]. This could be because performance-based cues have been shown to help the user understand the delivered cue.

A single-session closed-loop study provided visual feedback based on the patient’s own motion using eye-glasses and observed acute improvements in walking speed and stride length [84]. Two studies used treadmill walking with closed-loop visual cueing to demonstrate that people with PD could successfully follow the cues and improve the targeted gait parameters; one involved projection of target step length and uprightness cues (only one type of feedback was used at a given time) on the monitor in front of the treadmill [69] and the other projected visual cues (transverse lines) on the treadmill belt [70]. A few studies developed smartphone applications and utilized data from inertial measurement units to measure surrogates of current gait performance, which were obtained by calculating an average of the parameter over several steps, and provided feedback when the gait parameter was not in the target zone [85,86]. The feedback was provided to the user only when the gait pattern was insufficient and was referred to as “on-demand” feedback [86]. Of the closed-loop cueing studies listed in Table 1, two of them examined the immediate/acute effects of auditory cues in a single session using a wearable sensor system. The Ambulosono sensor system and the StepPlus system [87,88] were developed to provide auditory feedback to inform users when their current spatiotemporal gait parameters are out of a specified target range. Both systems [87,88] were designed for use by people with PD but have not yet been tested in people with PD. Preliminary results on a control population (a group of individuals without PD) showed improvements in stride length, stride length CoV, and cadence. The Armsense device, a portable device to measure arm-swing and provide tactile feedback, was tested in a single-session study on individuals with PD and demonstrated improvements in spatiotemporal gait parameters [89].

With mounting evidence suggesting greater gait and posture improvements as a result of closed-loop cueing training, a pilot study [69] was extended to assess the performance of cues on improving gait and posture in PD in a six-week training study [90,91].

Other long-term training studies using closed-loop visual and auditory cueing evaluated the effects of closed-loop cueing on a variety of gait parameters: gait speed [67,68,70,86,92,93], cadence [70,86], stride length [67,68,70,86], fall incidences [94], and other gait and dynamic balance measures [74,84,95] at follow-up and post-training. Only two of the long-term training studies used a wearable sensor-based, closed-loop system [86,95].

Some closed-loop training studies used augmented reality devices and game-based motion therapy for combinational cueing [72,75,77,84,96,97]. Results from these studies suggested that the closed-loop sensory feedback with or without long-term training was an effective non-pharmacologic intervention for gait and balance improvement in PD. The abovementioned studies involving virtual reality and game-based visual cueing have provided feedback to the user using monitors placed at the eye-level which may help people with PD to be upright at least while following the feedback.

The regular practice of being upright during the training and any sustained benefits may reduce the issue of stoopness experienced by people with PD. To date, only a few closed-loop studies [70,74,86,94,95] included a randomized control trial (RCT) research design to confirm that the gait and posture improvements observed are mainly due to the presentation of cues.

## 7. Discussion

### 7.1. Different Cueing Types May Engage Different Mechanisms

Findings from the literature indicate that both types of cueing (i.e., auditory and visual) result in improved gait and posture in individuals with PD. The hypothesized neural mechanism for external cueing, suggested by Morris et al. [92], bypasses the hypoactive basal ganglia-supplementary motor cortex (SMA) circuit by slightly altering the way the neural circuits control movement in individuals with PD [31,35,99]. In general, sensory cues are known to enable the dorsolateral pre-motor control system [30,32,63] which bypasses the SMA that is deficient in PD. Specifically, it has been suggested that auditory cues help in improving the temporal parameters, such as cadence and gait speed, and that external cues help because they are able to bypass the internal rhythm deficit associated with PD. Visual cues, on the other hand, are believed to enable the visual–cerebellar motor circuit that influences the spatial aspects of gait, such as step/stride length [71,82,92,100].

### 7.2. Effect of Disease Stage on Cueing Strategy

The effect of cueing in PD rehabilitation may depend on the stage of the disease and the type of dominant symptoms being experienced. The studies included in this review focused predominantly on individuals classified as Hoehn and Yahr stages II, III, and IV. For people in the early stage of disease severity, external cues can compensate for small deviations from their normal gait pattern thereby maintaining optimal gait quality and preventing deconditioning through training. Severely affected individuals with PD rely on external cues to compensate for deficits in the automatic control mechanisms (i.e., the ability to automatically generate normal stride length in a timely manner) thus improving gait and reducing the incidence of falls and freezing of gait [30,32,37,55].

### 7.3. Open-loop Cueing: Challenges and Limitations

The primary challenge in cueing, whether open-loop or closed-loop, is to present the cue in a manner that is informative, but does not have detrimental side effects on gait or balance. Despite numerous studies that demonstrated the benefits of cues on gait in PD, most of them did not investigate cues effects on balance control. Also, studies that specifically used cues to improve balance control in PD are very limited, and they were focused on improving posture during quiet stance, sit-to-stand, and dynamic balance maneuvers [101,102,103]. It is possible that the presentation of visual cues in the form of markers on the floor or on the treadmill belt [38,70,81] may further degrade posture and stability because it requires people with PD, who may already experience stooped posture, to look down.

Similarly, the use of auditory cues provided via earphones may reduce the awareness of environmental sounds which may make it unsuitable for use outside of a laboratory environment. This could be particularly problematic if sounds are provided continuously, i.e., with every step. Another major limitation associated with open-loop systems is that the user is required to detect any mismatch between the cue and their performance and decide how to respond in a manner that will get them entrained (in sync) with the cues. For auditory cues, the user might have to make a quick or a long-duration step in order to get in phase with the cues; for visual cues, the user might have to make a short or a long step in order to get in phase. Finally, although the literature on open-loop cueing in PD includes several studies that observed considerable improvements in spatiotemporal parameters, future studies along these lines could help to move the field forward by documenting how well users are able to follow the cues and by utilizing an RCT research design. Documentation of performance in following the cues could provide insight into the limitations of the cue presentation technique and could help to document the progression of learning throughout an intervention; the use of an RCT design would provide more reliable and actionable evidence for a decision to use a technique in the clinic.

### 7.4. Closed-loop Cueing: Challenges and Limitations

As with open-loop cueing, closed-loop strategies must also present information in a manner that is informative and does not have detrimental side-effects on gait. In addition, closed-loop paradigms must also measure/calculate the feedback parameter in real-time and, if it is to be useful outside of the laboratory, the entire system should be wearable and affordable. Setups that use motion capture systems in the laboratory or clinic are expensive and require travel and staff time. Treadmill-based systems pose limitations because some people with PD do not feel comfortable walking on a treadmill, whether that be at home or a facility with supervision. For these reasons, a low-cost wearable system that could readily be used on a daily basis during overground walking might be more widely accepted. However, there are technical challenges in measuring gait parameters from wearable sensors in real-time and conveying feedback in a manner that is safe and easy-to-use. Our group and others are working to develop low-cost, wearable systems for real-time feedback in home or community environments [87,104,105]. Once the technical development challenges are overcome, these systems will be evaluated for accuracy and safety and then clinical efficacy will have to be assessed in an RCT. These types of technologies have potential for widespread use, but they would require regulatory approval before commercialization and marketing.

## 8. Conclusions

Based on the review of the literature presented here, it is clear that cueing can be an effective component of locomotor therapy for people with PD who experience gait deficits. Rhythmic auditory cueing has been the most widely used technique, but it is most effective only in influencing the temporal parameters of gait. Visual cueing techniques have been used to increase spatial parameters, such as step/stride length, and to reduce step/stride length variability and asymmetry. Such improvements could have a high clinical impact, as they are important factors in gait and posture rehabilitation for people with PD. However, the usefulness of visual cueing techniques has been limited by challenges in presenting cues in a manner that is practical outside the laboratory and in a manner that encourages upright walking. To overcome the limitations of currently available techniques, several groups are developing unobtrusive wearable systems for closed-loop cueing to provide feedback of performance on a step-by-step or on-demand basis. These systems seek to improve locomotion during activities of daily living by providing feedback of gait and posture parameters that are often deficient in PD and by providing it in a way that can be readily used on a regular basis in the home or the community. Recent engineering developments have produced technology that is suitable for applications that require wearable sensors. Current challenges are to develop algorithms to interpret information from the sensors in real-time and to present it to the user in a manner that is intuitive, non-distracting, and actionable. Such advances that lead to technology for cueing that is effective, affordable, and wearable may enable adoption of these techniques by individuals with PD for use on a regular basis at home and in the community.

## Figures and Tables

**Table 1:** Summary of key findings of closed-loop cueing strategies that were included in this review.

Study	Intervention Type	Sensors; Feedback Mode	Outcome Measures	Study Protocol	Results	Limitations
Badarny et al. 2014 [84]	Visual	Wearable motion sensors; virtual reality based eye-glasses	Walking speed, stride length	Single-session study with cue and a follow-up evaluation (1 week later)	Increases in both walking speed and stride length, immediate effects and at follow-up	No control group; only assessed short-term effects
Jellish et al. 2015 [69]	Visual	Treadmill-based, video-based motion capture system and a feedback monitor	Step length, postural (back) angle measured during treadmill walking	Single-session study using multiple trials with and without cues	Increases in uprightness and step length	Utilized technology that is only available in research labs
Chomiak et al. 2019 [87]	Auditory	IMU sensors with a smartphone application-(Ambulosono sensor system)	Step length, walking distance, velocity, and cadence	Single-session study multiple trials	Evaluation of the sensor’s performance on healthy controls	Use of iPod Touch for feedback is not cost-effective and the system has not been evaluated on PD population
Bartels et al. 2018 [88]	Auditory	IMU sensors with a smartphone application	Stride length	Single-session study with multiple trials	Evaluation of the sensor’s performance on healthy controls	The system has not been evaluated on PD population
Young et al. 2014 [98]	Auditory-sonification of gait–swing phase	Video-based motion capture system with a smart phone application	Step length CoV	Single-session study in the lab with multiple trials	Reduction in step length variability	Utilized technology that is only available in research labs
Thompson et.al. 2017 [89]	Somatosensory	IMU sensors with a software application on the laptop and a vibratory device	Step length, lateral trunk sway, cadence, gait velocity, arm swing	Single-session study in the lab with multiple trials.	Increases in step length, arm swing magnitude, reduced cadence	Though somatosensory cues have been successful in helping with the rhythm of the movement, they are less effective in increasing the amplitude of the desired movement
Schlick et al. 2016 [70]	Visual	Treadmill-based pressure platform and video feedback monitor	Gait speed, stride length and cadence	Long-term training (5 weeks) at lab, RCT	Both the training and control group showed increases in gait speed and stride length post training, but sustained effects after 2 months were observed only in the case of feedback-based training	Small sample at follow-up because of attrition
Mirelman et al. 2016 [94]	Visual	Video-based motion capture system with virtual reality feedback	Fall incident rates	Long-term training (3 times/week for 6 weeks) at lab	Reduction in the rate of falls during the 6 month follow-up evaluation	No control group
Baskaran. 2017 [90]	Visual	Treadmill-based video-based motion capture system and a feedback monitor	Step length, postural (back) angle measured during treadmill walking	Long-term training (3 times/week for 6 weeks) at lab	Increases in uprightness and step length	No control group and a small sample size
Yang et al. 2016 [75]	Visual	Video-based motion capture system with virtual reality feedback	BBS, DGI, TUG test	Long-term training (2 times/week for 6 weeks) at lab, RCT	Increase in clinical score, BBS performance which was retained at 2 week follow-up	Small sample size
Van den Heuvel et al. 2014 [60]	Visual	Video-based augmented feedback system with treadmill and IMU sensors	FRT, BBS, UPDRS	Long-term training (2 times/week for 5 weeks) at lab, RCT	Improvements in balance scores in favor of the feedback system	Changes in scores were not statistically significant
Ginis et al. 2015 [86]	Auditory	IMU sensors with a smartphone application (CuPiD system)	Gait speed, cadence, stride length and stride length asymmetry	Long-term training (3 times/week for 6 weeks) at home, RCT	Increase in gait speed at post-training	Assessors were not blinded
Carpinella et al. 2016 [95]	Auditory and visual	IMU sensors and monitor for exercise therapy with a Gamepad (Gaming Experience in Parkinson’s Disease)	BBS and gait speed	Long-term training (3 times/week for 6 weeks) at lab, RCT	Increase in clinical score, BBS performance and retained effects at 1 month follow-up	Lack of online computation of gait measures and the use of technology that is only available in research labs
Frazzitta et al. 2009 [68]	Auditory and visual	Treadmill-based strain gauge and a visual feedback monitor	Stride length, gait speed	Long-term training (4 weeks) at lab	Greater increase in gait speed and stride length following treadmill-based cue training than with overground-based cue training	No control group and the study did not evaluate residual effect at follow-up
Rochester et al. 2010 [81]	Auditory, visual, and somatosensory	IMU-based rhythmical feedback system	Walking speed, step length, step frequency	Long-term training study for 6 weeks at lab	Increase in walking speed and step length with all cue types in both single and dual-tasking after training.	No control group
Espay et al. 2010 [96]	Auditory and visual	IMU sensors and a head-mounted display and headphones	Gait velocity, stride length and cadence	Home-based training for 2 weeks	Increase in gait velocity and stride length after training	No control group
Pompeu et al. 2012 [74]	Auditory and visual	Wii Fit games	UPDRS	Long-term training (2 times/week for 7 weeks) with exercise therapy at lab	Decrease (improvement) in UPDRS post-training and at 2 month follow-up evaluation	No control group

Inertial measurement unit (IMU), Coefficient of variation (CoV)), Unified Parkinson’s Disease Rating Scale (UPDRS), Berg balance score (BBS), Dynamic gait index (DGI), Functional reach test (FRT), Randomized controlled trial (RCT). The table includes only experimental studies and does not list the review articles mentioned in the text.
